# Bacterial pigments: A colorful palette reservoir for biotechnological applications

**DOI:** 10.1002/bab.2170

**Published:** 2021-05-02

**Authors:** Viviana Teresa Orlandi, Eleonora Martegani, Cristina Giaroni, Andreina Baj, Fabrizio Bolognese

**Affiliations:** ^1^ Department of Biotechnologies and Life Sciences University of Insubria Varese Italy; ^2^ Department of Medicine and Surgery University of Insubria Varese Italy

**Keywords:** bioactive compound, biotechnology, *E. coli*, expression systems, enzyme, gene expression, melanin, microbial metabolism, pigments

## Abstract

Synthetic derivatives are currently used instead of pigments in many applicative fields, from food to feed, from pharmaceutical to diagnostic, from agronomy to industry. Progress in organic chemistry allowed to obtain rather cheap compounds covering the whole color spectrum. However, several concerns arise from this chemical approach, as it is mainly based on nonrenewable resources such as fossil oil, and the toxicity or carcinogenic properties of products and/or precursors may be harmful for personnel involved in the productive processes. In this scenario, microorganisms and their pigments represent a colorful world to discover and reconsider. Each living bacterial strain may be a source of secondary metabolites with peculiar functions. The aim of this review is to link the physiological role of bacterial pigments with their potential use in different biotechnological fields. This enormous potential supports the big challenge for the development of strategies useful to identify, produce, and purify the right pigment for the desired application. At the end of this ideal journey through the world of bacterial pigments, the attention will be focused on melanin compounds, whose production relies upon different techniques ranging from natural producers, heterologous hosts, or isolated enzymes. In a green workflow, the microorganisms represent the starting and final point of pigment production.

**Abbreviations**
5‐ALA: 5‐aminolevulinic acid
5MPCA: 5‐methylphenazine‐1‐carboxylic acid
ATP: adenosine triphosphate
(B) Chl(s): (bacterio) Chlorophyll(s)
Bchl a: bacteriochlorophyll a
Bchl(s): bacteriochlorophyll(s)
Chl a: chlorophyll a
Chl(s): chlorophyll(s)
DNA: deoxyribonucleic acid
DOPA: dihydroxyphenylalanine
EFSA: European Food Standard Authority
US FDA: United States Food and Drug Administration
FMN: flavin mononucleotide
HPPD: 4‐hydroxyphenylpyruvate dioxygenase
LAB: lactic acid bacteria
LH: light harvesting
MRSA: *Staphylococcus aureus* methicillin resistant
NADPH: nicotinamide adenine dinucleotide phosphate
PBPs: phycobiliproteins
PCA: phenazine‐1‐carboxylic acid
PDT: photodynamic therapy
PSs: photosensitizers
PG: prodigiosin
proto IX: protoporphyrin IX
PV‐2: Poliovirus type 2
RC(s): reaction center(s)
ROS: reactive oxygen species
SARS‐CoV‐2: severe acute respiratory syndrome coronavirus 2
UP: undecylprodigiosin

## 1 INTRODUCTION

The term Phoenicians origins from the Greek “phoenix,” the red purple pigment extracted from mollusc shells to dye textiles^1^. Currently, microbiologists and biotechnologists, behaving as “modern Phoenicians,” focus their attention on the microbial world as a plentiful source of pigments, which are exploited by different industrial fields, including the alimentary, cosmetic, pharmaceutical, and environmental industry. Indeed, most prokaryotic microorganisms produce chemically different colored compounds, carrying out diverse physiological functions. Bacteria and Archaea are widespread and may inhabit extreme environments, including oceans, volcanos, saline, which constitute an endless reservoir of microorganisms producing bioactive colored compounds. In this scenario, the availability of plentiful sources for microbial pigments has a great potential for the discovery of new bacterial‐derived dyes^2^. As much as the attention for the environment sustainability is increasing, this natural and unlimited colorful palette represents an environmentally friendly source that can substitute synthetic analogue compounds. Bacteria as a source for biocolorants are preferred to other natural sources, including plants and fungi, because of their stability and availability for cultivation throughout the year^3^. As an added value, bacterial cultivation may follow the principles of the circular economy aiming at waste elimination. Exploitation of agricultural wastes, such as molasses, seeds, and peels, not only allows to address disposal and environment pollution issues, but also to grow biocolor producing microorganisms^4^.

The United States Food and Drug Administration (US FDA) and European Food Safety Authority (EFSA) have approved few bacterial pigments for the nutraceutical industry, such as astaxanthin and β–carotene from phototrophic bacteria, thus far^5^, ^6^. Indigo, one of the oldest pigments for dyeing textiles, especially denim, is currently obtained by chemical synthesis. As this strategy is based on fossil feedstocks, scientists propose a more environmentally sustainable biotechnological process that exploits microbial biocatalytic systems^7^. In this context, it is reasonable to predict that microorganisms will be even more investigated in the future with the intent to solve problems related to human and animal health.

The aim of this review is to focus the attention on the most investigated pigments produced by bacteria and highlight the link between their physiological role and the possible biotechnological applications. In this last regard, a more clear‐cut knowledge of the biological functions of pigment producers has, at least, two important implications: (1) it allows the discovery of potential applications in different biotechnological fields, and (2) it favors the development of appropriate strategies to implement theirproduction. Moreover, the discovery of the genetic determinants as well as of the regulatory circuits involved in the biochemical synthesis allow to choose the more appropriate biotechnological process with maximum yield of pigment production in the same producer, either in a heterologous host or in an in vitro system.

## 2 BACTERIAL PIGMENTS AND VISIBLE LIGHT IRRADIATION

**FIGURE 1:**
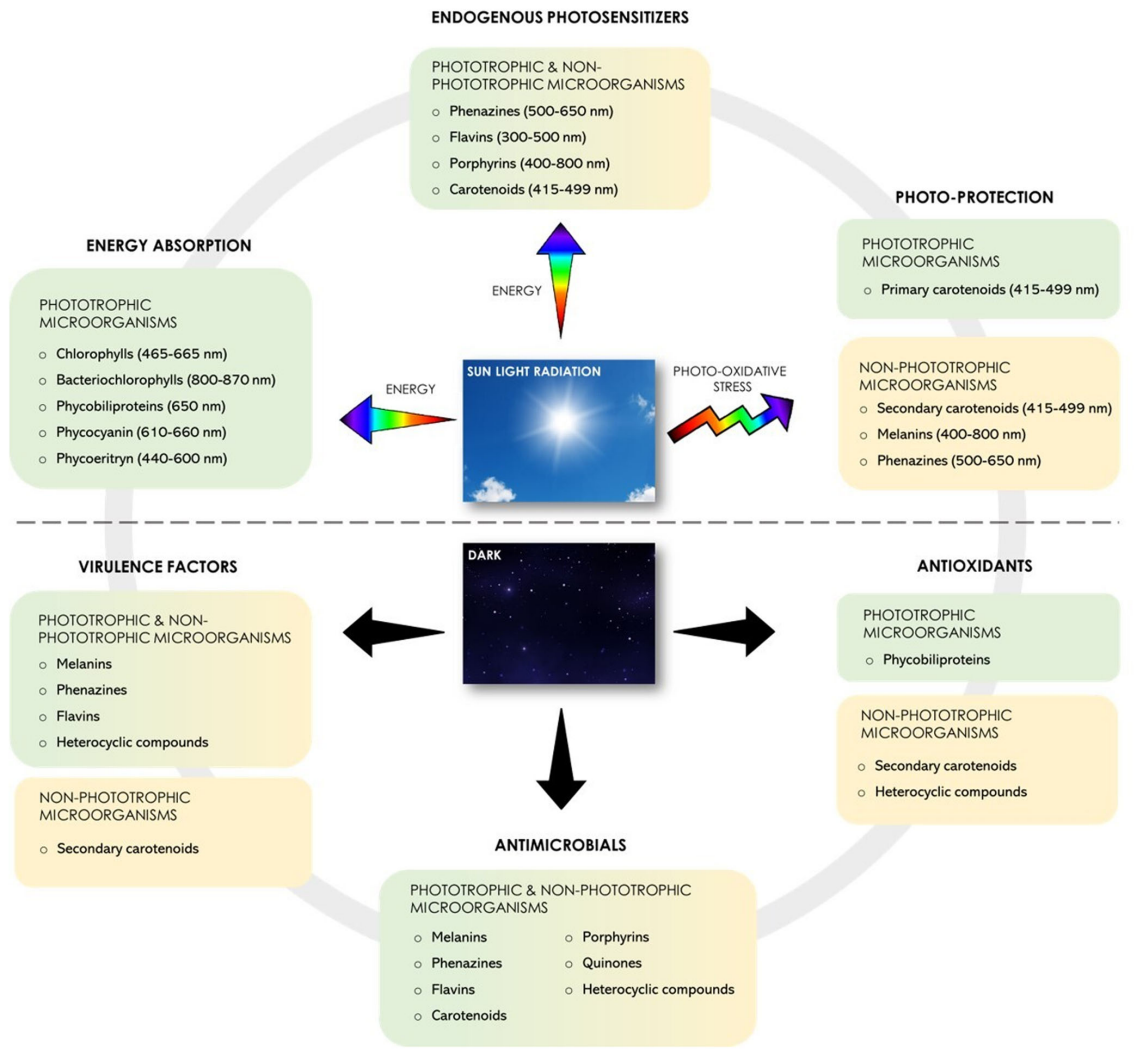
Schematic representation of the main physiological roles of bacterial pigments. The cited pigments produced by phototrophic and nonphototrophic microorganisms are involved in both light‐dependent and light‐independent mechanisms. Each pigment family involved in capturing sunlight radiation absorbs visible light in the specified range. In the dark, pigments increase the microbial fitness acting as virulence factors, antioxidants, antimicrobials

In the world of bacterial pigments, the sunlight plays a dual role by both permitting to perceive each pigment specific color and by taking part in their biological and ecological functions (Fig. 1).

1. The electromagnetic spectrum is the range of all possible wavelengths of radiation emitted by the sun, and visible light constitutes only a part of this spectrum. Bacterial pigments absorb only certain wavelengths of the visible light and reflect the color of each unabsorbed wavelength. In plants, algae and cyanobacteria, chlorophyll a (Chl a) has a unique and crucial role in converting light energy into chemical energy by absorbing wavelengths from both ends of the visible spectrum (blue and red), but not from green. As green is reflected, Chl appears green. Some pigments, such as melanin, absorb a broad range of the electromagnetic spectrum, conferring a brownish or black appearance to bacterial colonies on solid medium^8^.
2. Photosynthetic pigments, such as Chls (Chls) and bacteriochlorophylls (Bchls), carotenoids, ficobiliproteins, are fundamental for “phototrophic” bacteria, where visible light represents the energy source, alternative to organic or inorganic chemicals^9^. Pigments from light harvesting (LH) complexes capture photon energy and transfer it to reaction centers (RCs) pigments, where photochemical reactions promote an electron flow. Phototrophic or facultative phototrophic bacteria convert light energy into chemical energy either in the form of ATP, and/or of the reducing molecule, NADPH. Among these microorganisms, those defined photoautotrophic satisfy their carbon requirements exploiting ATP and NADPH to fix atmospheric carbon dioxide, whereas those defined as photoheterotrophic use environmental organic compounds. Bacteria often optimize their fitness by producing a mixture of pigments to absorb energy from a wider range of visible‐light wavelengths. Full access to sunlight is not achievable by all microorganisms since some bacteria grow underwater, where light intensity decreases with depth and the water absorbs certain wavelengths. The competition in the same ecological niche, among phototrophic organisms, drives their ability to capture light of specific wavelengths as a function of their own pigment reservoir as well as of light intensity^10^, ^11^.
3. In phototrophic bacteria, the activation of Chls and Bchls causes the development of photo‐oxidative stress that may be counteracted by accessory pigments. Almost 60 years ago, Griffith highlighted the antioxidant property of the carotenoid family. He reported damage in the carotenoid‐deficient facultative phototrophic *Rhodobacter sphaeroides* during photosynthesis in an aerobic atmosphere^12^. In this bacterial species, Bchl a reaches a triplet excited state (3 Bchl a*) during solar energy capture^13^ and carotenoids play an important role in protecting cells from photo‐oxidative stress induced by photodynamic reactions. In the so‐called “type I” photodynamic reactions, electrons flow from the excited photosensitizer (3 Bchl a*) to surrounding molecules and/or oxygen to produce radicals and/or reactive oxygen species (ROS), that is, O_2_^–^, H_2_O_2_, OH^.^. In “type II” photodynamic reactions, energy flows from 3 Bchl a* to oxygen (^3^O_2_), which is subsequently converted to singlet oxygen (^1^O_2_), a high reactive species that destroys biomolecules such as lipids, proteins, and DNA, causing cellular impairment and death^14^.
  In nonphototrophic bacteria, sunlight radiation may promote photo‐oxidative stress, and pigments, such as melanins and phenazines, may counteract this photodegradation^15^. In spore of *Bacillus subtilis*, melanin protects from UV irradiation^16^.
4. On the other hand, researchers highlighted the possible prooxidant role of other pigments, such as flavins and porphyrin derivatives. These compounds may act as photosensitizers and, upon activation with the appropriate light wavelength, increase ROS levels and ^1^O_2_, probably by photodynamic reactions type I and/or type II^17^. Even if this type of photosensitization is not desired in nature owing to the deleterious outcome in cells, the effects of endogenous photosensitizers has been recently investigated for the discovery of new disinfection procedure^18^.
5. Pigments playing a critical role in photosynthesis may have other uses in nonphototrophs bacteria. Many microorganisms live in dark places, impenetrable to sunlight radiation, such as deep sea, underground, and inside human and animal bodies. Many pigment families favor the adaptation of most microorganisms, independently from sunlight radiation. Among these, melanins, phenazines, chinones, flavins, heterocyclic compounds, and other pigments are secondary metabolites that are not essential for the producers to survive but are useful to increase bacterial health status under environmental stress conditions such as oxidative stress, microbial competition, and starvation^3^. Recently, Lubner^19^ focused the attention on interesting questions on the use of light in nonphotosynthetic biological systems. Indeed, light can also be used to activate a number of photosensory compounds and proteins designed to carry out tasks other than energy production^19^. Blue light has also been found to regulate several physiological processes such as metabolic pathways, motility, and virulence^20^. In both photosynthetic and nonphotosynthetic bacteria, the lateral gene transfer is suggested to be responsible for the production of pigments allowing to exploit new environmental and metabolic mechanisms^21^.

## 3 WIDESPREAD PHYSIOLOGICAL FUNCTIONS OF BACTERIAL PIGMENTS

As previously mentioned, pigments related to phototrophic metabolism, exploit sunlight radiation as a positive energy donor and may also counteract the subsequent induction of negative photo‐oxidative stress. Pigments that are not involved in phototrophic metabolism play different roles, which may be either linked, or not, to sunlight radiation. In this scenario, it is functionally important to distinguish between phototrophic and nonphototrophic pigments. In the following sections, the structure and function of the more widely investigated bacterial pigments are shown.

### 3.1 Photosynthetic pigments

In phototrophic bacteria, pigments are pivotal elements in the context of refined mechanisms underlying exploitation of solar radiation. Chls and BChls, carotenoids, ficobiliproteins, form the LH antenna and the RC of photosynthetic complexes in bacteria^22^, ^23^. The term “chlorophototrophs” indicate organisms that have photochemical RCs and perform Chl‐based phototrophy. Among these, green sulfur bacteria, green nonsulfur bacteria, purple bacteria, and heliobacteria are non‐oxygen evolving, whereas cyanobacteria release oxygen^24^. The last one played an essential role in the evolution of complex life, by contributing to transform a reducing early Earth environment into an oxygenated world^25^. In facultative phototrophic bacteria (i.e., *Rhodobacter* spp.), microorganisms can switch between energy sources, from light to chemicals, for survival. The formation of photosynthetic complexes is influenced by the light intensity in such a way that pigments are produced and are essential only under certain environmental conditions^26^.

The biodiversity of phototrophic bacteria, in terms of metabolism, habitat, environmental niche, growth conditions, depends also on the richness and variety of their pigments. Phototrophs exploit Chls and hundreds of carotenoids to utilize most of the solar radiation that reaches the earth, from the near‐UV (∼350 nm) to the near infrared (∼1050 nm)^23^. Each bacterial pigment allows the absorption of narrow ranges of the light spectrum. This diversity permits to improve bacterial health and overcomes the competition in the same environmental niche. The spatial arrangement of photosynthetic pigments is fundamental for producing energy, and bacteria have evolved diverse supramolecular antenna structures, for example, phycobilisomes in cyanobacteria, chlorosomes in green bacteria, or carotenoids–Bchls complexes as well as photochemical RCs containing various Bchls or Chls^23^.

#### 3.1.1 Chlorophylls and bacteriochlorophylls

**FIGURE 2:**
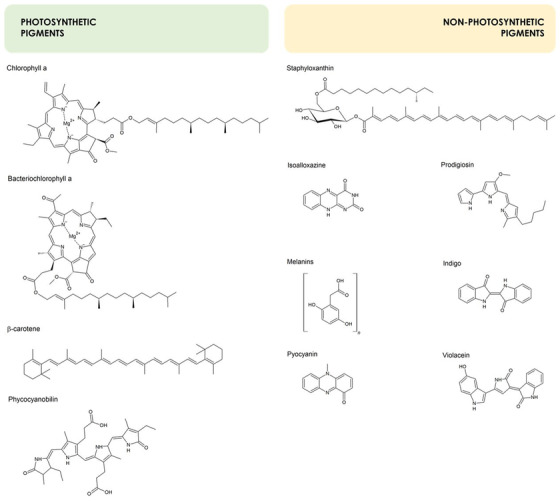
Chemical structures of photosynthetic and nonphotosynthetic pigments cited in the text. Chlorophyll a, bacteriochlorophyll a, β‐carotene, and phycocyanobilin were chosen as representative photosynthetic pigments. Among bacterial pigments not involved in photosynthetic processes, staphyloxanthin, isolalloxazine, melanins (shown as a polymer of homogentisic acid), pyocyanin, prodigiosin, indigo, and violacein were included

Chlorophylls are the more widespread photosynthetic bacterial pigments, known also as “pigments of life” involved both in LH and photochemistry^27^. Chls are a group of macrocyclic tetrapyrrole containing a central Mg^++^. A phytol moiety, consisting in a long chain of esterifying alcohols, influences the aggregation of Chls and, therefore, the interaction with the environment^28^. Many anoxygenic phototrophs possess Bchls that may contain a central Zn^++^ in the tetrapyrrole macrocycle. Different types of Chls (Chl a, b, d; divinyl‐Chl a and b; 81‐hydroxy‐Chl a) and/or Bchls (Bchl *a, b, c, d, e*, and *g*) collect and convert solar energy into chemical energy. The range of light that can be absorbed by (bacterio) chlorins for photosynthesis ranges from ∼350 to ∼1050 nm in the UV and near infrared. Their spectral absorption properties change depending on the substituents on the macrocycle and on the interaction with other Chls, carotenoids, proteins, and lipids. Although (B)Chls are different in their structure and spectral properties, their synthesis is based on a common precursor, protoporphyrin IX, and phototrophs share highly conserved enzymes responsible for most of the biosynthetic pathway. The metabolic precursor of all (B)Chls is protoporphyrin IX (Proto‐IX), which is also the precursor of heme and heme derivatives such as the bilin chromophores in phycobiliproteins (PBPs). Proto‐IX is synthesized from eight molecules of 5‐aminolevulinic acid (5‐ALA)^23^. Most Chls and Bchls are often embedded in Chl‐binding proteins, forming the antenna structures^29^. The spectral properties of the photosynthetic apparatus are determined by the arrangements of Chls and Bchls, depending on whether they are self‐assembled or organized within a protein scaffold, as well as by the chemical and spectroscopic properties of these pigments^30^. The chemical structures of Chl a and Bchl a are represented in Figure 2.

#### 3.1.2 Primary carotenoids

Primary carotenoid is the term used to specifically indicate carotenoids involved in photoprotection and LH in phototrophs. Carotenoids are polyisoprenoid, and according to their chemical composition, are classified as carotenes (C, H) or xanthophylls (C, H, and O) and absorb in the blue‐green range of the UV–visible spectrum,^31^. Among different, carotenoids, β‐carotene is orange, lutein is yellow, astaxanthin red‐pink, fucoxanthin green‐brown, β‐cryptoxanthin orange‐red^32^. Hundreds of carotenoids have been identified and their biosynthetic pathways involve the transformation of acetil‐CoA in C5 isoprenoid precursors that condense to C10, C15 and C20, C30 derivatives, to form C40 polyene chains of 8–13 alternate double bonds with benzene ring at one or both ends of the molecules^33^, ^34^. More recently, C50 carotenoids have been isolated from Antarctica heterotrophic microorganisms^35^. Aerobic anoxygenic phototrophic bacteria, such as heliobacteria, green sulfur and not sulfur bacteria, purple bacteria, use carotenes or xanthophylls to capture 400—500 nm light, which is then transferred to Chl, thus protecting from photo‐oxidative stress. Carotenoids neutralize ROS through an electron transfer that cause**s** the allylic hydrogen abstraction and addition reactions^31^. Furthermore, carotenoids counteract ^1^O_2_, by forming a reactive carotenoid molecule and the triplet nonreactive ^3^O_2_. In these conditions, the excitation energy dissipates through rotational and vibrational interactions, that is, release of heat, regenerating the original carotenoid molecule. The primary function of carotenoid pigments in phototrophs is to act as chemical buffers against photo‐oxidation of other cell constituents by Chl, thus conferring a high degree of immunity to endogenous photosensitization^36^. The chemical structure of β‐carotene is represented in Figure 2.

#### 3.1.3 Phycobiliproteins

PBPs represent a family of accessory pigments. Phycocyanin, phycoerythrin, phycoerythrocyanin, and allophycocyanin are the most investigated PBP. Each protein is formed by two different polypeptides (α, β), which probably derive from ancient gene duplication events and assemble as trimers (αβ)_3_ or hexamers (αβ)_6_. Each PBP contains multiple chromophoric bilin prosthetic groups, which confer extremely high absorbance coefficients to PBP. Phycobilins such as phycocyanobilin (*A*_max_ = 640 nm) (represented in Fig. 2), phycoerythrobilin (*A*_max_ = 550 nm), phycourobilin (*A*_max_ = 490 nm), and phycoviolobilin (*A*_max_ = 590 nm) are linked by carbon bridges, noncyclic tetrapyrroles lacking metal atoms in their structure^37^. Phycobilins are enzymatically synthetized from the precursor 5‐ALA, derived from glutamic acid, in a process requiring tRNA Glu. The condensation of two 5‐ALA to porphobilinogen is the first step of a pathway leading to hydroxymethylbilane, and then to uroporphyrinogen III. From this latter molecule, cyanobacteria produce protoporphyrin, the phycobilins precursor and Chls. PBPs are responsible for LH and transfer into photosystems with high efficiency^37^. In cyanobacteria, PBP absorb blue‐green light, in a wavelength range of the visible spectrum that cannot be absorbed by Chls. Indeed, cyanobacteria are able to colonize environments such as sea depths, which are rich in blue‐green light. Furthermore, PBPs are antioxidant agents like cyanobacterial carotenoids^38^.

### 3.2 Nonphototrophic bacterial pigments

Nonphototrophic bacteria produce a variety of pigments, whose role in the ecological environment has only be partially characterized thus far. The following paragraphs aim to describe the main features of a selected list of the most investigated nonphototrophic bacterial pigments.

#### 3.2.1 Secondary carotenoids

Secondary carotenoids refer to carotenoids which are not involved in phototrophic metabolism. Among nonphotosynthetic carotenogenic bacteria, *S. aureus* strains produce a membrane‐bound “secondary” carotenoid, known as staphyloxanthin (C_51_H_78_O_8_) (Fig. 2). Staphyloxanthin is a secondary metabolite, which is not necessary for *Staphylococcus aureus* growth, instead it is used by the pathogen to survive in infected hosts and to elude the immune system^39^. Liu et al.^40^ showed that a *S. aureus* mutant with disrupted carotenoid biosynthesis was more susceptible to oxidant toxicity, indicating that carotenoids may represent virulence factors. Furthermore, carotenoids can convert prooxidants metals, such as iron and copper derivatives, into harmless molecules, acting as metal chelators^33^. Recently, the zeaxanthin diglucoside was extracted from an endophytic *Pseudomonas* spp. strain. Fidan and Zhan^41^ suggest the potential use of this strain as a plant‐promoting strain for agricultural applications. The production of surface‐active compounds and carotenoid pigments by *Gordonia* spp. allows this group of microorganisms to grow under different conditions^42^.

#### 3.2.2 Flavins

Flavins are yellow pigments derived from isoalloxazine (Fig. 2), a tricyclic heterocycle containing oxygen and nitrogen. Among flavins, the main microbial pigment is riboflavin, known also as vitamin B2. Riboflavin biosynthesis occurs through seven steps catalyzed by enzymes encoded from five genes, organized in the *rib* operon (*rib*GBAHT)^43^. In a successive step, flavokinase RibC catalyzes the transformation of riboflavin and ATP into flavin mononucleotide (FMN), which is then transformed into various molecules by the enzymes RosB, RosC, and RosA, ultimately producing roseoflavin^44^. Riboflavin is involved in cellular metabolism as a structural component of the coenzymes, FMN and flavin adenine dinucleotide, playing key roles in redox homeostasis, protein folding, DNA repair, fatty acid β‐oxidation, amino acid oxidation^45^. Roseoflavin and toxoflavin are structural riboflavin‐analogs isolated from *Streptomyces* spp. and *Burkholderia* spp., respectively, showing antimicrobial activity^44^, ^46^.

#### 3.2.3 Melanins

Melanins are a heterogeneous group of pigments with an undefined structure, composed of polymeric compounds formed by oxidation and polymerization of phenolic, indolic, or homogentisic acid monomeric units (Fig. 2)^47^. Melanins absorb light from all the electromagnetic spectrum and are brown/black, but yellow‐reddish melanins have also been described^48^. The genetic background of melanogenesis in prokaryotic microorganisms has been investigated in several producers. In *Streptomyces antibioticus*, l‐methionine induces the expression of the *melC* operon that controls melanin production through the activity of an apotyrosinase^49^. Among melanin producer microorganisms, pyomelanin synthesis in *Pseudomonas aeruginosa* has been well illustrated and involves the conversion of l‐tyrosine in 4‐hydroxyphenylpyruvate through the action of the aromatic aminotransferase TyrB. Successively, 4‐hydroxyphenylpyruvate dioxygenase (HPPD) converts 4‐hydroxyphenylpyruvate into homogentisic acid, which undergoes oxidation to form acetic benzoquinone. This latter compound self‐polymerizes to produce pyomelanin^50^. Homologue key enzymes have been isolated from *Shewanella colwelliana*, *Vibrio cholera*, *Hypomonas* sp^51^. Recently, the production of pyomelanin in *Klebsiella pneumoniae*, *Alcaligenes faecalis*, *Enterobacter* spp., and *Vibrio splendidus* was shown to be linked to the activity of HPPD^52^, ^53^. *Marinomonas mediterranea* contains a laccase (*ppoA gene*) involved in melanogenesis through a two‐component system of signal transduction^54^. Melanin is an energy transporter and virulence factor with remarkable protective properties from UV irradiation and environmental stress^55^. Some melanin derivates have been isolated from endospore coats to protect from UV radiation^48^.

#### 3.2.4 Phenazine compounds

Many bacterial species produce phenazines, a group of nitrogen containing heterocyclic compounds with different properties depending on the type and position of substituent groups^56^. Even if *Pseudomonads* are the most investigated phenazine producers, other Gram‐negative and Gram‐positive bacteria produce phenazines such as *Sorangium, Brevibacterium, Burkholderia, Erwinia, Pantoea agglomerans*, and *Streptomyces*. In silico analyses highlighted that a biosynthetic operon core is required for the synthesis of the three‐ringed phenazine structure, which is probably shared among different genera by horizontal gene transfer^57^. Indeed, the pigmentation of *P. aeruginosa* is the result of the production of different phenazines such as pyocyanin, besides pyoverdine, pyomelanin, and pyorubin^58^, ^59^, ^60^, ^61^. The most studied phenazine is pyocyanin (5‐N‐methyl‐1‐hydroxyphenazine) (Fig. 2). Pyocyanin is a water‐soluble and nonfluorescent phenazine that changes color according to its oxidation state: it is blue in the completely oxidized state, and colorless in the reduced state^56^. Pyocyanin acts as an electron shuttle and can modify cellular redox state by altering electron flow patterns. In *P. aeruginosa* PAO1 strain, the presence of two quorum sensing regulated operons (*phzA1B1C1D1E1F1G1* and *phzA2B2C2D2E2F2G2)* leads to the synthesis of phenazine‐1‐carboxylic acid (PCA), which is subsequently converted to pyocyanin by a methyltransferase and a monooxygenase, encoded by *phzM* and a *phzS*, respectively^62^. Pyoverdine, a fluorescent yellow green siderophore, plays an important role in iron uptake^59^; pyorubin is a nonfluorescent red pigment helping in protecting microorganism from oxidative stress^61^. Pyocyanin and the other phenazines are virulence factors showing antimicrobial properties and conferring a selective advantage over other microbes in the natural environment. These pigments regulate cellular gene expression that trigger bacterial survival and biofilm formation^56^.

#### 3.2.5 Heterocyclic pigments

Heterocyclic compounds possess a cyclic structure with two or more different kinds of atoms. Among heterocyclic pigments, the so‐called prodigiosins (PGs) are red, whereas indigo and indigoidine are blue, and violacein is purple^3^. Three members of the PG family, PG (Fig. 2), undecylprodigiosin, and cycloprodigiosin hydrochloride, are characterized by a common pyrrolylpyrromethene skeleton^63^. *Serratia* spp., *Zooshikella* spp., and actinobacteria are the main producers of PGs. *Serratia marcescens* contributes to the biocontrol of plant diseases by inhibiting the growth of several phytopathogens. PGs confer the typical blood‐like appearance to starchy foods contaminated by *S. marcescens* ^64^.

Indigo, represented in Figure 2, is a dark blue heterocyclic pigment deriving from the degradation of indole, which represents a versatile bacterial messenger influencing bacterial physiology and virulence. Indole compounds are considered as interkingdom signaling molecule involved in the pathogenesis of human diseases as well as in the control of animal behavior^65^. Many bacterial oxidoreductases, such as naphthalene dioxygenase, biphenyl dioxygenase, phenol hydroxylase and cytochrome P450 hydroxylase, oxidize indole to indoxyl, which is further dimerized into indigoids. Indole is not the inherent substrate for these enzymes, which are crucial to catabolize energetic substrates. However, most of these enzymes are responsible for the conversion of indole into the nontoxic dye indigo, through an oxygenation reaction depending on the presence of electron‐donating cofactors. Indigo seems to be a cellular waste, rather than playing a physiological role in the bacterium life^66^.

Violacein, represented in Figure 2, is a bisindole produced by several Gram‐negative nonphylogenetically related genera and may be isolated from very different environmental niches, including marine, freshwater, and soil environments^67^. Its biosynthesis begins with l‐tryptophan and it is catalyzed by enzymes encoded by the *vioABCDE* operon^68^. This violet pigment is a secondary metabolite often associated to biofilm formation and its production is regulated by quorum sensing signaling. Violacein shows antibacterial properties against Gram‐positive bacteria, such as *S. aureus*, and displays toxicity toward bacteriovorus predators, such as *Caenorhabditis elegans* ^67^. The antioxidant activity of violacein has been described as involved in membrane defense against oxidative stress^69^, eukaryotic predation, and fungal diseases^70^, ^71^.

## 4 BIOTECHNOLOGICAL APPLICATIONS OF BACTERIAL PIGMENTS

A multidisciplinary scientific approach is fundamental for the development of modern biotechnology, whose progress is greatly influencing the economic world and the lifestyle of human beings. The United Nation Convention on Biological Diversity defines biotechnology as “any technological application that uses biological systems, living organisms, or derivatives thereof, to make or modify products or processes for specific use.”^72^ Bacterial pigments fit very well within this definition.

**FIGURE 3:**
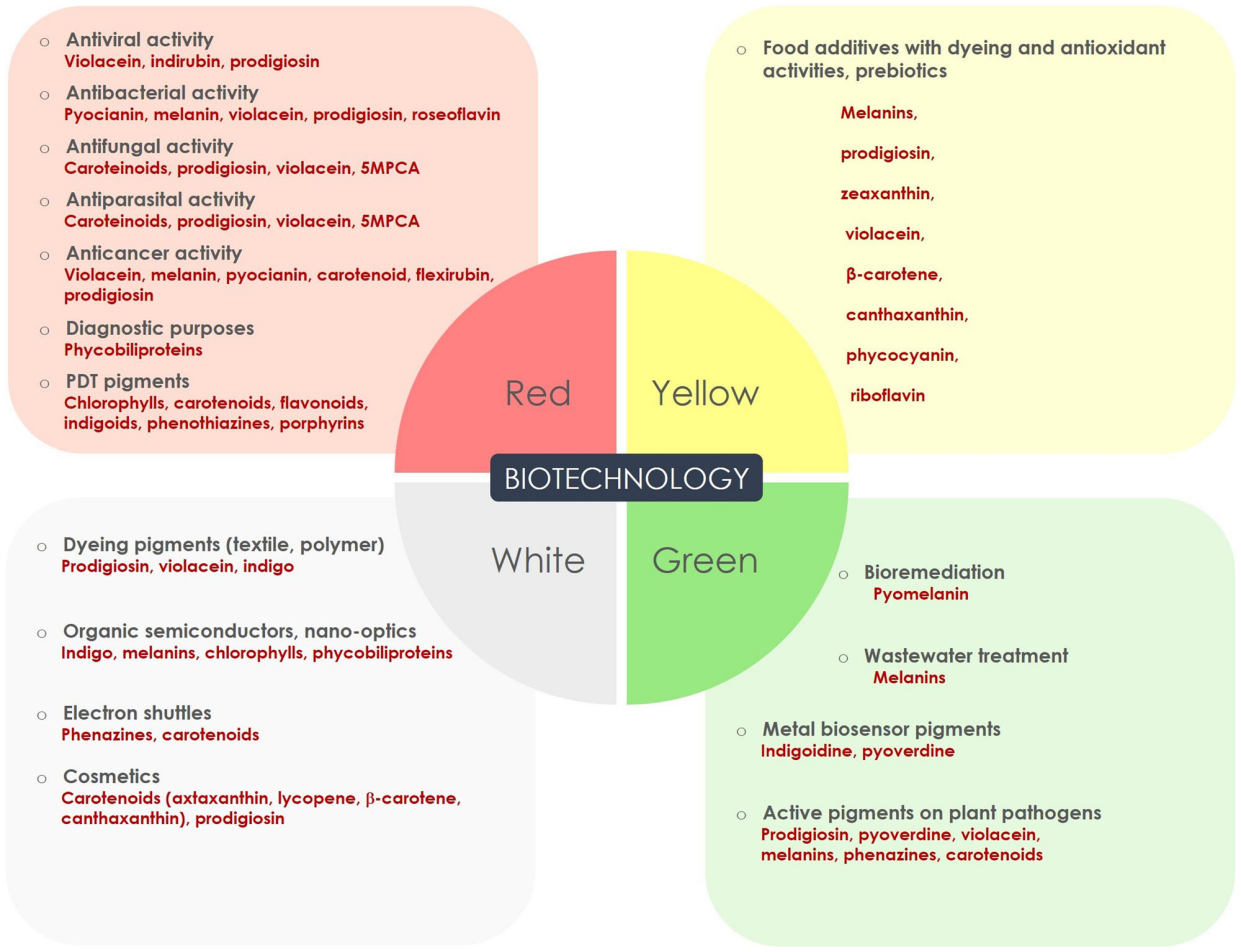
Applications of bacterial pigments in medical (red), alimentary (yellow), industrial (white), and environmental/agricultural (green) biotechnology. For each field the pigments cited in the text are depicted

An increasing number of studies and reviews report the great potential of bacterial pigments in different applicative biotechnological fields. An overview of the possible uses of pigments can be illustrated with the biotechnology “rainbow code,” which symbolizes different biotechnology branches with different colors. Thus, “red biotechnology” is for human health applications, “yellow biotechnology” is for food and nutrition, “white” indicates industrial biotechnology, and “green biotechnology” refers to agriculture, plant, and environment^72^. A synopsis of the potential applications of bacterial pigments in the different fields is summarized in Figure 3.

### 4.1 Bacterial pigments and red biotechnology

Red biotechnology aims to discover new drugs^72^. In these times more than ever, the discovery and emergence of new viruses, microbial superbugs and multidrug‐resistant infections and cancers, represent human life‐threatening issues. A study estimated that 10 million deaths due to antimicrobial resistance would occur every year after 2050. In addition, in the same year, cancer incidence will double as a consequence of population growth and ageing^73^. Furthermore, the current pandemic spread of SARS‐CoV‐2 is leading toward unpredictable and alarming scenarios. While biomedical research is principally aimed at obtaining an efficient vaccine, old and new drugs are administered to counteract the clinical manifestations of viral infection in different host tissues, comprising the respiratory, urinary, and cardiovascular systems^74^. Among putative antiviral drugs, bacterial pigments represent an unlimited reservoir of natural compounds to face new and old pathogens as well as cancer.

#### 4.1.1 Antiviral pigments

As new viruses are developing in humans consequently to spillover events, investigations on novel natural compounds with putative antiviral activity are compelling. Among heterocyclic compounds, violacein showed a weak inhibition of viral replication of Herpes Simplex Virus‐1, Poliovirus type 2 and Simian rotavirus SA11^75^. Indirubin, an indigoid pigment, which can be synthetized through flavin‐containing monooxygenase, showed antiviral activity in human bronchial epithelial cells H292 infected with influenza virus A NWS/33 and B/Lee/40, by reducing both the expression and production of the chemokine RANTES^76^. PG was efficient in decreasing the viral titers of *Bombyx mori* nucleopolyhedrovirus (BmNPV), an enveloped double‐stranded DNA virus with a high tropism for silkworm. PG was shown to behave as an inhibitor of DNA replication and transcription of BmNPV. Moreover, PG treatment was found to successfully prevent BmNPV‐mediated cell membrane fusion, which can block viral cell‐to‐cell transmission.^77^ Molecular docking analyses showed binding interactions of PG with the active sites of proteins from hepatitis B virus genotype B2, human immunodeficiency virus, and influenza A virus^78^. Indeed, these results suggest that prodiginines may represent a new group of antiviral compounds.

#### 4.1.2 Antibacterial pigments

The antimicrobial activity is often associated with pigmented secondary metabolites that control the growth of bacterial competitors and, thus, increase the fitness of bacteria in each environment. Although it is well established that pigments help the producer to eliminate other species of bacteria colonizing the same niche, a definite mechanism of action was hypothesized for few compounds^79^. Among phenazines, pyocyanin, which is responsible for *Pseudomonas* spp. blue‐green color, was formerly defined as colicin owing to its ability to inhibit *Escherichia coli* growth^80^. Pyocyanin conferred antimicrobial activity against other *P. aeruginosa* physiological competitors, such as *S. aureus*, *Staphylococcus saprophyticus*, and *Enterococcus faecalis* ^81^. Pyocyanin has been suggested to interfere with the cell membrane respiratory chain, thus impairing energy‐requiring, membrane‐bound metabolic processes, such as active transport into the cell^82^.

Melanin from *Pseudomonas balearica* has been proposed as a biocontrol agent of the phytopathogenic *Erwinia* ^83^. *Streptomyces davaonensis* and *Streptomyces cinnabarinus* produce roseoflavin, a promising broad‐spectrum antibiotic, inhibiting the growth of *S. aureus*, *E. faecalis*, *Streptococcus pyogenes*, *Listeria monocytogenes* ^84^. Violacein producers are generally sessile bacteria, which are constitutively more vulnerable to predation. This last observation lends support to the hypothesis that the purple pigment serves to accomplish a defensive mechanism^67^. Violacein extracted from *Chromobacterium violaceum* showed strong antibacterial activity against *S. aureus*, possibly via the disruption of the membrane integrity, as recently suggested^85^. A recent review reported the renewed interest in anti‐infective activities of the red pigment PG, against both Gram‐negative and Gram‐positive pathogens^86^. PG drastically alters the cell membrane integrity of *B. subtilis* and *E. coli* ^87^. Both heterocyclic pigments, violacein and PG, altered the permeability of the cytoplasmic membrane and, consequently, the physiology of the cell. Bacteria cell membrane is the cellular compartment mediating most of the functions that in eukaryotic cells take place in organelles. Alteration of membrane integrity impairs the proton gradient and ATP synthesis; consequently, some essential functions, such as solute transport into the bacterial cell, DNA, and peptidoglycan synthesis, are compromised. Pigments behave more as disinfectants than antibiotics, as the former have a broader spectrum of activity than the latter.

Recent investigations highlight the efficacy of the most promising pigments in killing multidrug resistant strains and inhibiting/eradicating pathogen biofilms. Violacein was active against *S. aureus* methicillin‐resistant (MRSA) strains^86^ and inhibited the biofilm formation of *Staphylococcus epidermidis*, an opportunistic pathogen that forms adherent communities on catheters causing chronic infections and sepsis in hospitalized patients^88^. PG inhibited the biofilm formation of *P. aeruginosa* by ROS production^89^. The combination of natural pigments with traditional antibiotics may represent a strategy to counteract the spread of superbugs. In this context, PG in combination with β‐lactamic antibiotics showed a synergistic effect against a MRSA strain^90^.

#### 4.1.3 Antifungal and antiparasitic activities

The most important families of bacterial pigments show antifungal and antiparasitic activities. Recently, yellow pigments, putatively belonging to the carotenoid family, showed antifungal activity against selected fungal pathogens of economic importance, such as *Sclerotium rolfsi* and *Rhizoctonia solani* ^91^. PG, combined with chitinase, inhibited the germination of *Mycosphaerella fijiensis* ^92^. Violacein was found to be effective against a number of plant and human pathogenic fungi and yeast species such as *Cryptococcus gastricus*, *Trichophyton rubrum*, *Fusarium oxysporum*, *R. solani*, *Aspergillus flavus*, *Penicillium expansum*, and *Candida albican*s^93^. Among phenazines, 5‐methylphenazine‐1‐carboxylic acid (5MPCA), was efficacious against pathogenic fungi, such as *C. albicans* ^94^.

At concentrations higher than those of pentamidine, violacein showed antileishmanial activity without remarkable side effects^95^. Antimalarial activity was observed for PG and violacein. The latter was active against chloroquine resistant strains of *Plasmodium falciparum* ^96^.

#### 4.1.4 Anticancer

Toxicity, adverse events, and resistance represent major issues for oncology chemotherapy. Several reports in literature are available to demonstrate the possible efficacy of bacterial pigments in overcoming these limitations, by influencing apoptosis or autophagy pathways in cancer cell lines. Interestingly, violacein selectively induced apoptosis in HL60 cells, a cancer cell line used as a model to study myeloid leukemia, but not in normal lymphocytes^97^. The black extracellular melanin, from *Streptomyces glaucescens* NEAE‐H, was cytotoxic against a skin cancer cell line^98^. The blue green pigment pyocyanin significantly inhibited human hepatoma cells and glioblastoma cells^99^, ^100^. Among carotenoids, the yellow pigment from *Streptomyces griseoaurantiacus* induced a significant cytotoxicity against cervical cancer cells with low IC_50_ ^101^. Flexirubin, a carotenoid from *Chryseobacterium artocarpi*, induced apoptosis in breast cancer cells MCF‐7 and the combination with silver nanoparticles showed synergistic effects^102^. PG showed strong anticancer and apoptosis effects on human cervical and laryngeal cancer cells^103^. Indeed, prodiginines are also attractive options because several multidrug resistance pumps, which can confer resistance to anticancer chemotherapy drugs, do not interact with them^104^, ^105^. The combination of PG with doxorubicin determined a synergistic effect in oral squamous cell carcinoma^106^. The mechanism of action of PG is actually under investigation. Wang et al.^107^ observed the ability of the red pigment to inhibit Wnt/β‐catenin signaling and reduce cyclin D1 levels. PG has been proposed to have therapeutic activity against advanced breast cancers.

#### 4.1.5 Diagnostic approaches

In the red biotechnology, bacterial pigments, behaving as fluorophores, may be exploited in diagnostic applications. Since 1989, the use of phycoerythrin has been suggested to evaluate the rate of peroxyl radical scavenging in human plasma^108^. Upon UV irradiation, PBPs may be used in flow cytometry and histochemistry^109^.

#### 4.1.6 Photodynamic therapy

**FIGURE 4:**
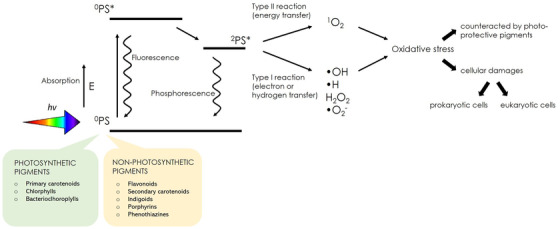
Application of bacterial pigments in four branches of biotechnology: medical (red), alimentary (yellow), industrial (white), and environmental/agricultural (green) applications. For each field, the pigments cited in the text are depicted

In the last twenty years, the photodynamic therapy (PDT) emerged as a promising treatment for both cancer and antimicrobial clinical settings. PDT is a technique based on the irradiation of dyes (photosensitizers or PSs) to induce, in the presence of oxygen, the production of ROS and ^1^O_2_, which are toxic for eukaryotic and prokaryotic cells, respectively (Fig. 4). In this context, Staron et al.^110^ suggested to search for possible PSs among different bacterial pigments, including Chls, carotenoids, flavonoids, indigoids, phenothiazines, and porphyrins. The photosynthetic pigments, Chls and Bchls, have been proposed as excellent natural photosensitizers. In particular, Bchl a was modified to photodynamically treat a preclinical model of colon cancer^111^, conjugates of Bchl a and Chl a were found to be effective against bacteria, such as *E. coli* ^112^. Yoshii et al.^113^ observed that human skin melanoma cells were damaged after neoxanthin, fucoxanthin, and siphonaxanthin irradiation. These carotenoids share energy states above ^1^O_2_, and this feature seems to confer photosensitizing properties in contrast to other carotenoids, such as β‐carotene, which share energy state below ^1^O_2_ ^113^. Indeed, most pigments possess many conjugated double bonds that can absorb visible light, behaving as ideal PSs. On these bases, the exploitation of bacterial pigments in PDT for antitumor and antibacterial applications deserves much attention. It must be said, however, that many pigments have constitutive antimicrobial and/or antitumor activities regardless of light irradiation, as previously described.

### 4.2 Bacterial pigments and yellow biotechnology

The use of colorants in food and alimentary industry mainly relies on synthetic compounds, which are characterized by average low costs of production and high chemical stability. The occurrence of possible adverse events, such as allergenicity, toxicity, and carcinogenicity, makes the production of natural alternatives a main goal in the so‐called “yellow biotechnology.”^114^ Although, the US FDA, the EFSA, and the World Health Organization have imposed safe dosages for the use of food colors^115^, some confusion in the distinction between natural and synthetic additives still persists, and a legal definition for “natural compound” has not been adopted yet^116^. Many colored substances deriving from living organisms, such as plants, fungi, and bacteria, are devoid of the negative drawbacks of the chemical counterparts and display antioxidant, antibacterial, or anticancer activities. Several bacterial pigments show a potential use as food grade additives. For example, the interest in β‐carotene is increasing due to its use as a colorant in food industry in concentrations between 2 and 50 ppm, in juices, drinks, butter, margarine, and cheese. It is also used as a nutritional supplement, being the precursor of provitamin A, and the involvement of vitamin A in the vision process is well known. Furthermore, hypovitaminosis A represents one of the major nutritional problems in least developed regions of the world^117^. The extraction of β‐carotene from microbial feedstocks could meet the increasing request of the world food market that is based on synthetic derivatives. However, the production process of this pigment is hampered by solubility, stability, melting point, and low bioavailability issues. The delivery of carotenoids through polymeric nanocapsules may overcome such drawbacks^118^. The yellow zeaxanthin, from *Flavobacterium* spp., was suggested as an antioxidant colorant in food^119^ and as an additive in poultry feeds^120^. Canthaxanthin from *Bradyrhizobium* spp., an orange/deep pink pigment, displayed potent antioxidant properties^121^. *Aphanizomenon* spp. belonging to cyanobacteria produce the blue pigment phycocyanin, which is used in food and beverage industry^116^. Most studies have been done in *Spirulina* spp. cyanobacteria with a high phycocyanin content reaching the 20% of its dry weight. However, the sensitivity of phycocyanin to heat treatment results in precipitation and fading of the blue color and limits its use in food. The addition of sugars and polyhydric alcohols, safe for consumption, could overcome this issue and stabilize proteins^122^.

The red pigment PG, produced by *S. marcescens*, represents a further source replacing synthetic dies used in food industry. Although PG offers several limitations including solubility and short stability upon exposure to pH, light and high temperatures, an alternative delivery system was suggested. Kappa‐carrageenan and maltodextrin were used to encapsulate the red pigment that was proposed in a spray‐dried formulation, as a coloring agent in yogurt, milk, and carbonated drinks. The moisture content, particle size, and color intensity of PG encapsulated were optimized for food consumption^123^.

Violacein, produced by *C. violaceum*, is a powerful antioxidant stimulating mucosal defense mechanisms. There are interesting data on the ability of violacein to interfere with the composition of the rat gut microbiota. The oral administration of the violet pigment to rats in low (50 μg/ml) and high (500 μg/ml) doses for a month influenced the composition of the microflora: Bacilli and Clostridia were abundant in the low violacein dose, while Bacilli followed by Clostridia and Actinobacteria were present as the major components for the high violacein dose^68^. If the observed changes will be evaluated as beneficial to the host, the use of bacterial pigments as prebiotics could represent an important add value in the food industry. Melanin pigments are used as food colorants and nutritional supplements, and soil bacteria grown on fruit waste have been used in food industry, as proposed by Tarangini and Mishra^124^. Kiran et al.^125^ proposed a very interesting use of melanin from actinobacterium *Nocardiopsis alba* MSA10. The synthesis of silver nanoparticles mediated by melanin was developed for their potential incorporation in food packaging materials and antimicrobials for stored fruits and foods. However, the efficacy of melanin–silver nanoconjugates on the shelf life of packed food products needs to be investigated^125^.

Riboflavin is accepted in dairy products, drinks, and baby foods^126^. Recently, the EFSA Panel on Additives and Products or Substances used in Animal Feed concluded that the use of riboflavin produced using *B. subtilis* KCCM‐10445 as a feed additive for all animal species poses a risk for the spread of viable cells and antibiotic resistance determinants^45^. A valid alternative is the use of lactic acid bacteria (LAB) that can be used in the food industry owing to the acknowledged status of qualified presumption of safety^127^. As *Lactobacilli* produce riboflavin, the development of vitamin B2 enriched probiotic food was investigated. Riboflavin‐hyperproducer LAB were obtained by the selection of variants carrying few mutations in *rib* operon that codifies enzymes involved in vitamin B2 biosynthesis. Among the obtained mutants, *Lactobacillus fermentum* PBCC11.5 was used to fortify bread. Bread produced using the inoculum of yeast and *L. fermentum* PBCC11.5 led to an approximately twofold increase of the final vitamin B2 content^127^. This strategy permits to overcome the spread of resistance determinants.

Though much promising in perspective, the use of natural colors in “yellow biotechnology” is challenging, both in term of cost production and product stability. When used in confectionary manufacturing, for example, natural pigments are as much as 20 times more expensive than synthetic compounds^128^. Another drawback of natural pigments is due to their sensitivity to environmental conditions such as light, temperature, pH, and oxygen concentration^129^. In general, the low bioavailability, solubility, and stability of bacterial pigments such as carotenoids can be overcome with the help of nanotechnology formulations. Micro‐encapsulation or production of nanoemulsions with surfactants, allow a more effective food coloring, with consequent cost reduction^130^. Given the great potential in health benefits displayed by natural pigments, many challenges are waiting to be addressed at the biotechnological level for the identification of new producing species and the construction of hyper‐producing strains. The possible identification of low‐cost fermentation substrates together with the increase of natural color shelf life will ensure the availability of substances which shouldn't be considered any longer as mere food additives.

### 4.3 Bacterial pigments and white biotechnology

Different bacterial pigments find applications in industrial production.

The phototrophs possess molecular complexes with defined optical response regions that pave the way for photonic materials based on biopigment assemblies. Pale suggested the use of bacterial Chls in nanophotonics, for instance, in organic solar cells, light emission diodes, and lasers^131^. For example, a solar cell manufactured from this material would be able to function even in cloudy days^131^. Similarly, the PBPs involved in bacterial photosystems LH, could be exploited in optics to assembly efficient light‐trapping devices for capturing solar energy under low light^37^. Carotenoid pigments isolated from the UV‐resistant Antarctic (red) bacterium *Hymenobacter* spp. and (yellow) *Chryseobacterium* spp. belong to the xanthophyllin family and represent a promising tool as photosensitizers in the production of dye‐sensitized solar cells technology. Sunlight photoactivation of purified carotenoids form the photoanode and deliver electrons to a titanium‐coated conductive glass. A redox electrolyte fills the space between electrodes and fulfils the role of regenerating the oxidized dye. This technology is still at the first stage of research and development and more efforts should be addressed to the identification of new biomolecules producing microorganisms^132^.

Furthermore, various carotenoids such as astaxanthin, lycopene, *β*‐carotene, and canthaxanthin are being commercialized to some extent and may find applications in cosmetics, thanks to their antioxidant properties^133^.

The bright red pigment PG from *Vibrio* spp. and *S. marcescens* was used to dye fibers such as nylon, acrylics, cotton, and silk. Though dyeing performances mostly varied with the fiber nature, colors were maintained upon variation of external conditions such as washing, perspiration, and rubbing^134^. PG could be a good candidate for coloration of polyolefins, such as polyethylene ultrathene. The pigment suspension from *S. marcescens* strain 9986 was introduced gradually up to rolled polymer sheet for the equilibrium coloration of the dyed stuff without spraying in the air^135^. In the cosmetic industry, PG showed to increase by 20–65 % the sunscreen protection factor of dermatological creams. The addition of PG to extracts of *Aloe vera* leaf, and *Cucumis sativus* fruit increased of one order of magnitude the protection factor^136^.

In a similar way, violacein, from *C. violaceum*, was used to color pure cotton, pure silk, rayon, and polyester. Notably, fiber dyeing was obtained either by simple fabric dipping into the dye solution or by boiling with bacterial cells; the color intensity varied with the dipping time and temperature^137^. Interestingly, an important aspect of marine bacteria belonging to the genus *Pseudoalteromonas* producing violacein in the crude extract, is their intolerance, and death, at human body temperature, suggesting their safety for industrial purposes^138^. Violacein is a component of several cosmetic products, displaying both rapid and prolonged contact with the skin, airways, or mucous membranes, such as antiperspirants, lipsticks, eye makeup^139^. Violacein from *Pseudoalteromonas* sp. (DSM 13623) was proposed for economical use in large amounts for consumer and environmental‐friendly products, especially in textile and toy industries^140^.

Indigo is one of the oldest textile dyes for popular blue denim, originally prepared from plant material and nowadays chemically synthesized from fossil feedstocks. In the perspective to develop a more sustainable and environmental‐friendly biotechnological productive process for this popular dye, a recent review reported an overview of the various microbial enzymes, which can produce indigo, highlighting the advantages and disadvantages of each biocatalytic system^141^. Besides naturally occurring enzymes, development of protein engineering for indigo production represents a promising and important resource. However, a large‐scale industrial biotechnological indigo production is not available, yet^141^. Besides textile industry, indigo was used for the construction of sustainable electronic devices. Indigo is an unusual organic semiconductor, featuring a highly planar, but relatively small cross‐conjugated, π‐electron system. This pigment undergoes rapid and reversible oxidoreduction processes, thus favoring the charge flow for biotransistors and inverters setup^142^. Similarly, melanins display a semiconductor like behavior for efficient dissipation of electromagnetic energy, such as heat^143^. In general, melanins are attractive natural polyphenolic compounds with broadband absorption in the UV–visible spectrum and, for this reason, can find many biotechnological applications^144^. Fresh melanin seems to be the product of proto‐molecules organization within onion‐like nanostructures. Much interest is devoted to the proposed porphyrin‐like tetramer structure, where the exposed nitrogen atoms in the core allow metal ion binding and the creation of larger molecules with high potential energy storage, usable in alternative energy batteries^145^, ^146^, ^147^. The presence of catechol residues and basic aminoacids inspired the production of adhesive films and/or nanoparticles via oxidation in moderate basic aqueous solutions or hydroalcoholic solutions. In the initial phases of the oxidative reaction, monomers or small aggregates can coat surfaces, whereas, when the reaction proceeds, the inherent size increases owing to polymerization, giving rise to the precipitation of nanoparticles, resembling natural melanin granules^148^, ^149^. The presence of hydroxyl‐, carboxyl‐, and quinone functional groups renders melanin able to bind and retain metal ions, although with variable binding affinity. This has been applied in the industrial treatment of waste waters contaminated with Pb(II), Cu(II)m, Cd(II), and Zn(II)^144^.

Phenazines are a class of soluble pigments produced, among others, by *P. aeruginosa* and are characterized by electron transfer properties^150^. This attitude seems to be exploited not only by producers, but also by other bacterial species, making these compounds a “collective good” to be used as an electron shuttle^151^. Phenazines are a promising tool in biofuel cells, where electrons generated by microbial consortia are not directly transferred to cognate receptors but, instead, are diverted to an electrode with production of electrical energy^152^. Interestingly, though redox conditions in microbial fuel cells are disadvantageous for aerobic bacterial species, *P. aeruginosa* becomes dominant in bacterial communities. On the other hand, constant oxidation of the electron shuttle phenazine causes overproduction of the dye concomitant with the enhancement of the bacterial concentration^153^.

### 4.4 Bacterial pigments and green biotechnology

Bacterial pigments and/or pigmented bacteria may also be used for “green biotechnology” applications in the agricultural bioremediation fields.

In agriculture, bioagents are preferred to chemical pesticides as they can be more selective and safer than chemical insecticides. Bacterial pigments represent an arsenal of compounds potentially useful for this aim. An insecticide containing violacein was effective in preventing plant mycosis, such as grass pythium blight, sclerotinia stem rot, bean sprout seedling blight, and plant parasitic nematode diseases such as watermelon *Meloidogyne* spp. diseases^140^. The potency of PG was proved against *Drosophila* larvae^154^, *Aedes aegypti*, and *Anopheles stephensi* ^155^. The previously described antiviral activity of PG against *B. mori* nucleopolyhedrovirus^77^ could be exploited to inhibit virus propagation and counteract deleterious effect on sericulture. A possible oxidative stress caused by PG seems to be involved in the toxic effects against *Microcystis aeruginosa* cells. Downregulation of gene transcription and cell lysis may represent a promising tool for combating *Microcystis* blooms and consequent environmental pollution^156^. Another interesting agronomic use of bacterial pigments concerns the insecticidal crystal proteins produced by *Bacillus thuringiensis*. As a melanin producer mutant of this bacterium protected the protein from UV radiation damage, the authors indicated that it could be useful for the industrial production of light‐stable, environmental‐ friendly insecticides^157^.

Phenazines produced by *Pseudomonas chlororaphis* display beneficial properties on plant roots, as the redox potential enables phenazines to cope with ROS generated by drought stress^158^. Importantly, *P. chlororaphis* represents a model suitable for whole‐cell application, as it displays low toxicity in humans. The growth of the plant pathogen *R. solani* is inhibited by phenazine derivatives produced by *Burkholderia cepacia*, whereas PCA showed activity against the hyphae of the pathogen *Botrytis cinerea* ^159^. As this latter microorganism causes great losses in the whole production chain of strawberries, grapes, and tomatoes, PCA may represent a promising natural remedy for post‐harvest control^160^. Pyoverdine produced by *Pseudomonas putida* strain B2017 also displayed antifungal activity. As this bacterial species does not produce HCN, pyocyanin, biosurfactants, or toxic metabolites, it may represent a promising biocontrol agent, without hazardous effects on nontarget organisms^161^.

Bacteria producing pigments can also be used as biofertilizer. Among phototrophic microorganisms, purple nonsulfur bacteria produce pigments such as carotenoids (e.g., spirilloxanthin, rhodopin, okenone, rhodopinal) that, together with vitamins and other plant growth‐promoting substances, counteract environmental stress and contribute to plant benefits^162^.

In bioremediation field, metals persisting and accumulating in the environment pose a threat to human health and ecosystems because they cannot be degraded or destroyed. As the methods commonly used for their removal are expensive and resource intensive, new green technologies are necessary^163^. Due to the metal affinity and high adsorption capacity of melanins, melanogenic bacteria can be used for bioremediation purposes. Nanoparticles obtained from the humic compound pyomelanin, purified from *Pseudomonas stutzeri* or *Azotobacter chroococcum* could bind Hg(II), Cu(II), Cr(VI), and Pb(II), showing promising potential in ion sequestration from polluted aqueous environments^164^, ^165^. Iron‐ and copper‐functionalized pyomelanin was used for trivalent and pentavalent arsenic removal from contaminated wastewaters. Interestingly, the system could be re‐used after ion reloading upon arsenic removal from melanin^166^. The release of radionuclides poses risks to ecosystems and requires innovative technologies for disposal of these substances and mitigation their detrimental effects. Turick et al.^167^ showed that, in uranium contaminated soils, tyrosine addition enhanced melanin production by indigenous microorganisms, resulting in uranium stable sequestration for up to 13 months. The fluorescent siderophore pyoverdine is commonly produced by *Pseudomonas* sp. strains and it is involved in iron homeostasis. Its ability to bind divalent ions, such as Cu(II) in soil matrices suggested a possible use in regulating copper photo‐availability in vineyard topsoil. Copper containing pesticides are not completely banned and are still widely used as fungicidal compounds with possible drawbacks on plant photosynthesis inhibition. The possible use of pyoverdine producing bacteria could represent a promising strategy to promote copper phyto‐extraction^168^.

Gu and Cheung^169^ observed a phenotypic change in the bacterium *Vogesella indigofera* upon exposure to environmental Cr^6+^ ions, which inhibited the routine production of the blue dye indigoidine. In this view, the microorganism could be used as a biosensor for specific metal detection in polluted areas, with a putative detection sensitivity between 200 and 300 μg/ml. The same pigment seemed to protect the organism during growth in cold water (below 15°C) and could be exploited as a cryo‐preservative additive^170^.

The treatment of waste waters represents a further green application of bacterial pigments. In the study of Gustavsson, the production of melanin was obtained in *E. coli* expressing a recombinant tyrosinase on external cell surface. This system proved to be very efficient in pharmaceutical contaminants removal from waste waters and a rapid regeneration of melanin matrix was obtained by simple pH adjustment^171^.

In this context and as previously reported, it is essential to highlight that the use of bacterial pigments is better than synthetic dyes that are not environmental friendly. The latter offer some limitations such as the requirement of hazardous chemicals and the disposal of hazardous wastes in the ecosystem. Furthermore, the process to obtain bacterial pigments can be managed in order to limit the impact on the environment. To this end, various agricultural products and byproducts such as corncob, sugarcane bagasse, grape waste, jackfruit seed, corn steep liquor, wheat substrates, and cassava were successfully used as growth medium for the cultivation of bacteria to produce pigments^172^. Thus, the bioprocess to obtain bacterial pigments and their exploitation in different application fields are part of green technology.

## 5 BIOTECHNOLOGICAL STRATEGIES TO PRODUCE BACTERIAL PIGMENTS

Different strategies can be developed to exploit bacterial pigments for biotechnological purposes. Once the pigment is identified, the best candidate among bacterial producers must be chosen. Indeed, the knowledge of the genetic background can drive the choice for the best producer. Microorganisms become biological machineries for pigment production, and it is essential to get a complete picture of the genetic determinants codifying the enzymes involved in pigment biosynthesis and the related regulatory network. Biotechnologists can choose between in vivo or in vitro strategies to obtain bacterial pigments. The first techniques use whole cells as factories of pigments synthesized in the cytoplasm and in some cases released in the extracellular environment. The in vitro approach resorts to one or more isolated enzymes.

A tentative roadmap can be outlined as follows:

- In the physiological context, the yield of metabolite production by the producer is highly dependent on environmental conditions. Wild strains are prone to produce pigments at low concentrations. The optimization of culture conditions in laboratory settings can increase the yield of pigment production, both in batch and in fermentation processes.
- Genetic engineering approaches may be used for the improvement of natural producer strains. Site directed or random mutagenesis, focusing on genetic determinants or regulators, lead to the selection of hyperproducer strains.
- The choice of a heterologous host carrying selected genes or operons can overcome the issues related to those natural producers that are unsafe and/or difficult to cultivate.
- The preparation of one or more isolated enzymes involved in the synthesis of pigments represents an alternative choice for in vitro production of microbial pigments.

### 5.1 Melanin pigments as an example of biotechnological resource

**FIGURE 5:**
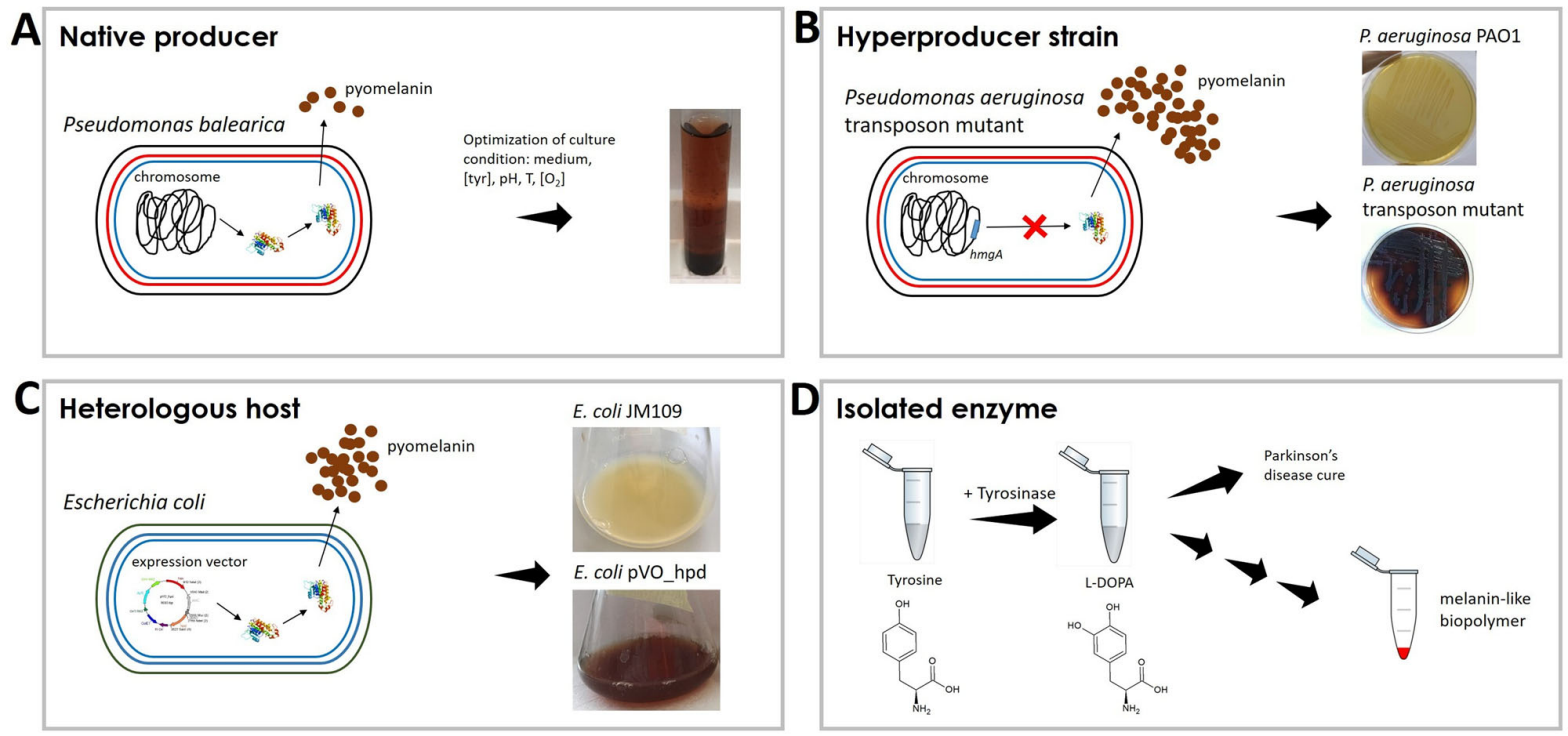
Schematic representation of four different biotechnological strategies used to produce pyomelanin. The *Pseudomonas balearica* strain was grown under different cultivation parameters to optimize the production of pyomelanin (panel A). A pyomelanin hyperproducer strain was obtained in *Pseudomonas aeruginosa* PAO1 upon knock out of *hmgA* gene codifying homogentisate‐1,2‐dioxygenase and involved in tyrosine catabolism, as represented in panel B. In panel C, *Escherichia coli* cells overexpress *hpd* gene codifying 4‐hydroxyphenylpiruvate dioxygenase from PAO1 strain. This approach represents the use of a heterologous host to produce large amounts of pyomelanin for biotechnological purposes. The chance to produce pyomelanin can be pursued through tyrosinase as isolated enzyme. Tyrosine is transformed into L‐DOPA that, in turn, can lead to the synthesis of melanin‐like polymers

To outline different strategies that exploit microbial cells, it is useful to focus the attention on one family of pigments. Among pigments, melanins are the most versatile for the high variety of potential applications. Melanin polymers represent a family of pigments widely distributed among prokaryotic and eukaryotic domains, even if with different chemical structures. As previously described, bacterial melanins may be used in different biotechnological branches, such as “red” (antimicrobial activity), “yellow” (antioxidant in food/feed), “green” (control of phytopathogen and bioremediation), and “white biotechnology” (bioplastics, optical lenses). Biotechnological strategies, both in vitro and in vivo, may be chosen to produce pyomelanin, (Fig. 5) as described herein.

### 5.2 Optimization of culture conditions for native producers

A sample of soil, seawater, and vegetable can be the source of unknown and promising melanin producers. Melanin production in vitro is easily noticeable by medium darkening. Batch cultivation in appropriate environmental conditions can improve the bioproduction of melanin. A *P. balearica* strain was isolated from the marine green alga *Ulva lactuca*, and grown in a semisynthetic medium containing l‐tyrosine, as sole carbon source, producing 29 mg/L of melanin (Fig. 5A). The addition of yeast extract enhanced the production of melanin up to 110 mg/L. Different temperatures (20–45°C) and the medium pH (pH 5.0–9.0) influenced the yields of melanin production, with the temperature range of 30–37°C, and a pH ∼8 representing the optimal conditions^83^. A more recent study reported the production of pyomelanin by marine *P. stutzeri* BTCZ10 after 180 h of growth in tyrosine basal broth (∼50 mg/L), yielding lower concentrations with respect to the former study^173^. The importance of this topic emerged in a recent review by Pavan et al.^174^ who sorted different melanin producers by melanin yield (g/L) and considered the parameters affecting pigment production, such as the addition of tyrosine and metal ions, the substrate chosen as source of carbon and energy, and the biotransformation time. The review outlines the add‐value of applying statistical analyses to increase pigment production. Optimization of pigment production results from the combination of analyses, performed before and after biotransformation, with the adjustment of critical growth parameters, such as pH, temperature, and agitation^174^. Tarangini and Mishra^124^ inoculated a nutrient agar with a garden soil sample to isolate different melanin‐producing colonies. Among these, the 16S rDNA sequencing identified a new species, *Bacillus safensis* ZJHD1‐43, which was cultivated in fruit waste and produced a high yield of melanin (∼7 mg/ml). Interestingly, the combination of two statistical approaches, central composite design and response surface methodology, optimized the production of melanin considering two critical parameters (pH and temperature)^124^.

### 5.3 Natural or artificial selection of pigment hyperproducer strains

*P. aeruginosa* produces different type of pigments, such as pyomelanin, pyocyanin, pyoverdin, and pyorubin. In their environmental niches, mutant isolates, that are able to produce high amounts of pigments, fit better than wild‐type strains. For example, melanogenic clinical isolates of *P. aeruginosa* regularly isolated from cystic fibrosis patients were more competitive compared to wild‐type parents^175^. In chronic infection isolates, the pigmentation originates from the accumulation of homogentisic acid, due to large chromosomal deletions that cause the lack of *hmgA* gene encoding homogentisate‐1,2‐dioxygenase. The inactivation of *hmgA* results in the secretion of homogentisic acid, which autoxidizes and self‐polymerizes to form pyomelanin^60^. Single point mutations occurring in this gene impair the function of the dioxygenase causing the accumulation of pyomelanin^176^.

The inactivation of *hmg*A gene can be pursued by in vitro molecular approaches, through random or site‐directed mutagenesis. A transposon bank was constructed in the model microorganism *P. aeruginosa* PAO1, and, among ∼2000 mutants screened on LB agar, one showed red–brown colonies and a gentamycin cassette interrupted the *hmgA* gene (Fig. 2B). The FT‐IR spectroscopy analysis of the supernatant from the mutant culture minimal medium, supplemented with 5 mM tyrosine, was compatible with pyomelanin^15^.

### 5.4 Pigment production in heterologous hosts

In different natural contexts, melanin produced by opportunistic pathogens plays the role of virulence factor. Thus, the exploitation of melanogenic bacteria for biotechnological applications poses some issues related to safety. Furthermore, wild strains grown in vitro do not reach high amount of biomass, necessary for scaling up processes. Genetic engineering offers different strategies to overcome these limits and it improves the yield of pigment production. In particular, the expression of key enzymes in a heterologous host is a good strategy. *E. coli* represents an optimal host because it does not produce melanin. *E. coli* W3110 expressing the tyrosinase coding gene (*melA*) from the nitrogen‐fixing *Rhizobium etli* CFN42 was used to transform tyrosine into melanin^177^. Chavez‐Bejar developed an engineering process by cloning a mutated gene form of *melA* from *R. etli* in *E. coli* in order to direct the carbon flow from central metabolism into the L‐tyrosine biosynthetic pathways. To this end, an *E. coli* strain lacking the sugar phosphotransferase system and tyr R repressor was obtained. However, the ability to grow on glucose was recovered by replacing the native promoter region of *galP* in the chromosome with the strong *trc* (*trp*‐*lac*) promoter. Furthermore, key enzymes of the l‐tyrosine pathway were overexpressed as feedback inhibition versions. The result was an engineered process of melanin production, relaying on glucose as carbon source, which greatly reduced the production cost with respect to l‐tyrosine as raw material^178^.

The cloning of *hpd* coding for 4‐hydroxyphenyl pyruvate dyoxigenase from *P. aeruginosa* PAO1, conferred *E. coli* JM109 the ability to produce pyomelanin^179^. Furthermore, the cloning of the genetic determinant under the control of an inducible promoter permits to turn on the enzyme expression, and, consequently, the pigment production at the chosen time. The administration of arabinose 0.1% increased threefold the production of pyomelanin in *E. coli*, as the transcription of the *hpd* gene was controlled by the *pBAD* promoter which, in turn, is regulated by araC gene product. Upon addition of arabinose, repression is relieved and AraC regulator contributes to maintain the promoter in a transcription‐proficient conformation, thereby permitting gene expression (Fig. 5C)^179^.

### 5.5 Isolated enzymes

An auspicious technology to produce the bioactive pigment melanin is represented by the use of purified bacterial tyrosinases. These copper containing monooxygenases catalyze the *o*‐hydroxylation of tyrosine (monophenol) to 3,4‐dihydroxyphenylalanine or DOPA (*o*‐diphenol) and its subsequent oxidation to dopaquinone. The *o*‐quinone can be transformed into melanin through a series of nonenzymatic reactions in which molecular oxygen acts as the oxidant species^180^. Five classes of prokaryotic tyrosinases have been identified, thus far, which differ in domain organization and in the necessity of auxiliary proteins (caddie proteins) for correct folding and protein activity^180^. Ren et al.^181^ describe the expression in *E. coli* of the tyrosinase gene from *Verrucomicrobium spinosum* and the identification of optimal cultural conditions for soluble and active protein production both in batch and fed‐batch systems^181^. Protein engineering led to expression and purification of a C‐terminal deletion of the enzyme with inherent increase in catalytic activity (from 30 to 100‐fold)^182^. A C‐terminal domain is reported in about 98% of mushroom tyrosinases, where enzymes are synthesized in a latent isoform activated by proteolytic cleavage. Notably, expression of the engineered tyrosinase isoform leads to a fully active enzyme, which, like *Rhizobium etli* tyrosinase, does not require copper chaperone for proper folding. *Verrucomicrobium spinosum* tyrosinase shows specific activity toward L‐tyrosine and L‐DOPA, but Molloy reported that genetically engineered isoforms of the enzyme from *Ralstonia solanacearum* show improved catalytic efficiency on d‐isomers^183^. Enzymatic immobilization of melanin on plastic surfaces allowed to apply tyrosinases catalytic properties for nerve growth. Moreover, melanin shows bacteriostatic activity and may be useful in preventing microbial contamination^180^.

As reported above, l‐DOPA is an intermediate in melanin production deriving from monophenolase activity (EC 1.14.18.1) of tyrosinases on l‐tyrosine, which is of high therapeutic value as a first line drug for Parkinson's disease treatment. The possibility to exploit the activity of immobilized tyrosinases in the presence of reducing agents, such as ascorbate, which prevent production of dopaquinone and melanin, may represent a challenging industrial approach for l‐DOPA production. In this perspective, isolated tyrosinase shows a double edge activity in the production of both bioactive pigments and pharmacologically relevant intermediates.

## 6 CONCLUSIONS

The increasing demand for ecofriendly and biodegradable supplies urges to the production of natural colorants. Most of living bacteria produce different pigments that increase their fitness and survival under physiological and/or stress conditions. Thus, prokaryotic biodiversity represents a colorful natural palette to exploit. Furthermore, it is desirable to increase the number of cultivable species, also from extreme environments, with the dual advantage to ameliorate our knowledge on the microbial world and to expand the availability of pigment sources.

Bacteria are the natural source of many pigments and represent the biotechnological tool to produce them. Indeed, the use of microorganisms to produce colorants is commercially and economically promising because of the ability to control growth conditions and to ensure renewability. Microorganisms are easily cultivated and propagated, and unlike vegetables, microbial growth is not influenced by seasonality. Furthermore, according to the modern circular economy, waste substrates can be used for bulk production. Pigment extraction from high quantity of biomass continuously growing in bioreactors is usually a simple process. The boost in biotechnological techniques offers many strategies to optimize pigment production, from natural producers and heterologous hosts to isolated enzymes in vitro.

The review highlighted several issues related to the biotechnological exploitation of bacterial pigments. For example, the use of pigments in the pharmacological, food, and feed fields may be difficult owing to their low solubility and the enormous progress in nanotechnology science is helping to discover new delivery systems to overcome this limit. Moreover, several pigment producers and/or the heterologous hosts pose a threat because of their virulence arsenal or antibiotic resistance, respectively. The efforts of researchers should be aimed at selecting microorganisms lacking any “dangerous” determinant. The engineering of safe probiotics could give a boost to develop the bacterial pigment world.

Furthermore, nowadays, there is a gap between the potential market and the patents available for bacterial pigments. It could be beneficial to create a world *in silico*‐platform collecting information on microorganism species and their pigments (chemical structure, spectral absorbance, putative physiological role, production pathways, genetic determinants, and control). The final user, that is, a biotechnological company, could be allowed to find the compound fitting with the specific commercial demand as well as the researchers with the related expertise. The optimization of bioprocesses, even if protected by corresponding patents, could be shared in the same platform, thus enriching the entire scientific community. In conclusion, the best applications for each bacterial pigment could be found to improve the life quality of human being.

## CONFLICT OF INTEREST

The authors report no potential conflict of interest.
